# Regulation of Interactions with Sliding Clamps During DNA Replication and Repair

**DOI:** 10.2174/138920209788185234

**Published:** 2009-05

**Authors:** Francisco J. López de Saro

**Affiliations:** Laboratorio de Ecología Molecular, Centro de Astrobiología (CSIC-INTA), 28850 Torrejón de Ardoz, Madrid, Spain

## Abstract

The molecular machines that replicate the genome consist of many interacting components. Essential to the organization of the replication machinery are ring-shaped proteins, like PCNA (Proliferating Cell Nuclear Antigen) or the β- clamp, collectively named sliding clamps. They encircle the DNA molecule and slide on it freely and bidirectionally. Sliding clamps are typically associated to DNA polymerases and provide these enzymes with the processivity required to synthesize large chromosomes. Additionally, they interact with a large array of proteins that perform enzymatic reactions on DNA, targeting and orchestrating their functions. In recent years there have been a large number of studies that have analyzed the structural details of how sliding clamps interact with their ligands. However, much remains to be learned in relation to how these interactions are regulated to occur coordinately and sequentially. Since sliding clamps participate in reactions in which many different enzymes bind and then release from the clamp in an orchestrated way, it is critical to analyze how these changes in affinity take place. In this review I focus the attention on the mechanisms by which various types of enzymes interact with sliding clamps and what is known about the regulation of this binding. Especially I describe emerging paradigms on how enzymes switch places on sliding clamps during DNA replication and repair of prokaryotic and eukaryotic genomes.

## INTRODUCTION

Central to the organization of the enzymatic machinery that replicates and repairs DNA in cells are the sliding clamp proteins, like the prokaryotic β-clamp and the eukaryotic PCNA (Proliferating Cell Nuclear Antigen) [1, 2]. These ring-shaped structures can interact with a large number of enzymes (See [3, 4] for recent updates) and orchestrate their activities during DNA replication and repair. Sliding clamps are uniquely designed to meet three critical needs in DNA synthesis. First, they tether DNA polymerases to the template, thus providing them with high processivity. Second, over the immense length of chromosomal DNA, sliding clamps are recruiting devices that provide a binding surface to target enzymes to active centers of DNA synthesis and repair. Finally a third, less understood function, point to their involvement as molecular beacons of newly replicated DNA, thus acting as signals to postreplicative repair systems or the higher-order regulatory apparatus that coordinates cell cycle progression.

**Fig. (1):**
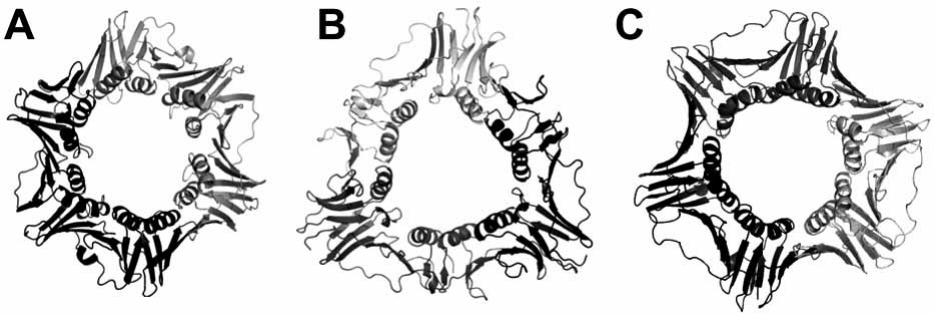
Crystallographic structures of representative sliding clamps. **A.** The dimeric prokaryotic β-clamp (2POL). **B.** The homotrimeric clamp from a bacteriophage RB69 (protein gp45) (1B77). **C.** The homotrimeric eukaryotic clamp PCNA (1SXJ).

Protein clamps are typically homodimeric or homotrimeric structures, like the β-clamp or PCNA, respectively, with the monomers binding each other in a head-to-tail fashion and completing a circle (See Fig. **1**) [5]. Recently heterotrimeric clamps have been found in archaea associated with replicative polymerases [6], and in higher organisms involved in checkpoint, like the RHR or 911 complex (Rad9-Hus1-Rad1) [7, 8]. Remarkably, across all life forms and despite limited or no sequence between them, sliding clamps are always composed of six domains with a characteristic fold [9]. A continuous layer of pleated sheet structure is the backbone of the ring, and a set of 12 α-helices line the inside of the clamp. Sliding clamps have two distinct faces with very different electrostatic properties [10]. The major site of interaction of clamps with partners is *via *a hydrophobic pocket between the two domains of each monomer (in PCNA) or domains 2 and 3 (in the β-clamp). Contacts have been also observed on the interconnector loop (only in PCNA) or the extreme C-terminal peptide of the clamp.

Since sliding clamps are co-factors of numerous proteins involved in the metabolism of DNA, interaction with sliding clamps can be a critical point of regulation of the activities of these enzymes. For example, processivity is increased up to 100-times when DNA polymerases are bound to sliding clamps and therefore changes in the affinity of the interaction could be a way by which the activity of the polymerase is regulated. Indeed, as described later, an internal switch within the DNA polymerase III complex in *E. coli* modulates the affinity with the β-clamp and this is the mechanism by which the polymerase regulates its processivity. Alternatively, in eukaryotic replication machineries the modification of either the sliding clamp or the binding surface of the partner enzyme often serves as a regulatory mechanism. Thus, ubiquitylation and sumoylation of PCNA have been described as transient modifications that serve to switch polymerases during the DNA damage response. Phosphorylation of PCNA-binding domains has been observed in numerous proteins involved in DNA replication, such as the cell-cycle regulator p21, Fen1, RFC, Pol δ and DNA ligase and this modification directly affects the interaction. Thus, the process of engagement and disengagement with sliding clamps may be a way of controlling protein function.

Regulation of sliding clamp binding is important in view of the fact that their concentration in the cell is likely to be limiting if compared to the concentration of proteins that bind to them, especially if it is assumed that only DNA-bound clamps are ‘active’. It has been estimated that there are only about 200-300 β-clamps in a *E. coli* cell. In eukaryotes PCNA expression is itself regulated and its levels are cell-cycle dependent. It seems unlikely that each enzyme uses its own sliding clamp exclusively, but rather that individual sliding clamps are trafficked by various proteins, either concurrently or sequentially. Numerous *in vivo* studies using microscopy and fluorescence have shown that PCNA clusters in regions of the chromosome that are being actively replicated, and that it co-localizes with polymerases and DNA repair enzymes [11, 12].

In the last decade we have learned that proteins use similar ways of interacting with sliding clamps but that use distinct mechanisms in the regulation of these interactions. In what follows I will review our knowledge of the structural details of how sliding clamps interact with their ligands, as provided by crystallography and biochemical studies, and then analyze in turn each of the emerging themes of regulatory mechanisms outlined above.

## BINDING AND AFFINITY OF PROTEIN LIGANDS TO SLIDING CLAMPS

Most proteins interact with clamps *via *residues in their N- or C-terminal regions [13] although internal binding sites are also critical in many cases. In prokaryotes the β-clamp consensus motif QLxLF has been recognized but is often poorly conserved and seems to be absent in a number of β-binding proteins [14, 15]. On the other hand, the consensus motif QxxLxxFF is present with minor variations in most PCNA-binding proteins [3, 13]. Since the proteins that harbour these motifs belong to many disparate structural families, these short sequences probably reflect convergent evolution for sliding-clamp binding.

The first detailed analysis of the binding surface of a clamp protein and its partner was that of the complex of human PCNA bound to a 22-residue C-terminal peptide of the cell-cycle inhibitor p21 [16, 17]. The p21 peptide uses a extensive array of contacts in binding to PCNA, interacting with the interconnector loop, the hydrophobic pocket and C-terminal residues of the clamp [17]. The C-terminus of p21 is disordered in solution and, in addition to PCNA, binds to a number of other ligands, including calmodulin and the oncoprotein SET [18, 19]. Relative affinities to these various ligands could be regulated by phosphorylation of the C-terminal domain of p21 [20].

The structure of a 11-residue C-terminal peptide of phage RB69 DNA polymerase in complex with its cognate clamp, the gp45 protein, has also been reported [21]. Although the interacting surface is much less extensive than in the case of p21, the peptide targets an analogous hydrophobic crevice on the clamp. Attachment *via *a flexible peptide allows the polymerase to remain tethered to the clamp while alternating between the spatially distant polymerizing and exonuclease modes of DNA binding. Another example of a polymerase interacting with its clamp has been recently provided by the structures of the C-terminal, ‘little finger’, domain of *E. coli* DNA polymerase IV and the β-clamp [22]. As with p21 and RB69, hydrophobic residues at the extended C-terminal peptide of Pol IV (in this case an ‘LGL’ motif) insert themselves in the surface pocket between domains 2 and 3 of the β-clamp. A distinctive feature observed in the Pol IV-β-clamp structure is a second binding surface that localizes to the interface between the two clamp monomers. A more recent crystal structure of Dpo4, the homologous Pol IV of *Sulfolobus solfataricus*, bound to its cognate clamp has given support to the idea that bound sliding clamp ligands often adopt highly flexible conformations [23].

A number of highly revealing studies of the enzymes involved in Okazaki fragment processing have shown multiple conformations of enzymes bound to PCNA. The interaction of Fen1 to PCNA has been studied using peptides [24] and the whole protein [25], indicating that the binding site is complex and consisting of extensive contacts near the interconnector loop and the C-terminus of PCNA. The binding site contains a hinge region that gives the complex high flexibility, and is highly reminiscent of that found in the case of p21 bound to PCNA [25]. The structure and dynamics of the interaction between *Sulfolobus solfataricus* DNA ligase and the heteromeric PCNA present in organism has been described [26], and that of a peptide of yeast ligase I (Cdc9) and PCNA [27].

Yet another class of structural insights has been provided by sliding clamps complexed to clamp loaders like the γ-complex or RFC. Clamp loaders are pentameric structures that are designed to bind an unloaded sliding clamp (i.e., not topologically bound to DNA) and, in an ATP-dependent reaction, break the ring and place it around DNA at specific sites [9]. The structure of the *Escherichia coli* δ subunit of the γ-complex complexed to a monomeric β-clamp shows that δ binds to β *via *two sites: first, an internal sequence (69-QAMSLF-74) that resembles the β-binding motif found in a number of other proteins interacts with the hydrophobic pocket between domains 2 and 3 of the clamp; the second contact is between α4 of δ and a loop on the β-clamp which as a result becomes restructured [28]. These contacts are important for opening and loading of the ring, and additional contacts of other subunits of the pentameric γ-complex have been demonstrated (See below) [28, 29]. On the other hand, biochemical studies have shown that in yeast RFC at least two subunits, RFC1 and RFC3, bind PCNA tightly [30]. These RFC subunits, like δ, bind PCNA *via *an internal sequence and contains conserved hydrophobic residues that have been predicted to interact with the hydrophobic pocket on the ring [30].

Pentameric clamp loaders are highly conserved, reflecting an ancient mechanism of interaction and handling of the sliding clamps [9]. In archaeal organisms the homologous RFC contains only two types of subunits, RFC-L and RFC-S, with a stoichiometry of 1:4 [31]. The crystal structure of a 11-residue C-terminal peptide derived from *Pirococcus furiosus* RFC-L bound to *Pfu*PCNA has been reported, showing an arrangement very similar to peptides bound to other clamps [32]. An extended region of acidic residues adjacent to the consensus binding box is important for binding to the clamp [33].

In general, although interaction with the hydrophobic pocket often represents a strong anchoring site used by clamp loaders and clamp-interacting proteins, other interactions at distinct sites on the rings are usually observed. Detailed studies of the affinity of ligands for the sliding clamps have been performed using peptides of the binding protein as models [15, 18, 34, 35]. However, the *in vivo* and functional relevance of these studies is often difficult to interpret due to the differences in affinity expected depending on whether DNA is present, or if the sliding clamp is topologically bound to DNA or not.

Two main themes emerge from the structural studies of sliding clamp complexes. Firstly, all complexes studied to date (in viral, archaeal, bacteria or eukaryotic systems) interact with the hydrophobic pocket on the same side of PCNA or the β-clamp. This often involves the two main hydrophobic and/or aromatic residues present in the sliding clamp motifs. Secondly, clamp binding is often highly flexible and the various dynamic conformations are dependent on DNA structure, which probably ultimately regulates the affinity of the interaction.

Crystallographic studies have shown us insightful close-ups of the interactions between clamps and their partners. In what follows I will describe the knowledge, mainly biochemical, of the processes and the context in which these dynamic interactions take place.

## LOADING OF CLAMPS ON DNA

Sliding clamps are opened and placed on DNA in ATP-dependent reactions by multisubunit complexes named clamp loaders [9]. The biochemistry of these enzymes has been studied in considerable detail for the case of the phage T4 gp44/62, *E. coli*’s γ-complex, and archeal and yeast RFC complexes [36-38]. Despite some distinct peculiarities of each clamp loader, the mechanism of loading of the clamp is very similar in all cases and it involves conformational changes in the clamp loader that couple ATP binding to binding to the clamp in solution, and ATP hydrolysis to its loading and release on a DNA primed site [9].

In *E. coli* the γ-complex (consisting of the γ_3_δδ’ subunits) first binds the β-clamp in solution through strong contacts with the δ subunit. However, this only happens when three molecules of ATP have been bound by the γ-complex [28]. The δ subunit binds to the β-clamp *via *a α-helix (α4) which undergoes major conformational changes involving rotation of four hydrophobic residues [28, 29]. In the absence of ATP α4 of δ interacts strongly with the δ’ subunit (*via *residues Trp61 and Phe62) and the β-clamp binding residues (Leu73 and Phe74) are sequestered on the surface of δ, unable to bind to the β-clamp. However, upon ATP binding, residues Trp61 and Phe62 loose their interaction to δ’ and Leu73 and Phe74 are projected and exposed, binding directly on the hydrophobic pocket of the β-clamp [28, 29]. Upon binding to primed DNA and hydrolysis of ATP, the process is reversed and the clamp is released already topologically bound to DNA.

Therefore the strong interaction of the clamp loader with the β-clamp is regulated tightly by conformational changes on the main interacting component, the δ subunit, which depend on ATP binding and hydrolysis and recognition of DNA structure by the clamp loader. Analogous conformational changes are thought to operate in the case of the archaeal [39] or the eukaryotic clamp loaders [30, 36]. Phosphorylation of human RFC by CamKII [40] and cyclin-dependent kinases has been reported [41], in both cases leading to a reduction in its affinity for PCNA. The detailed mechanism and consequences of phosphorylation of RFC are not clear, but they indicate that modulation of the affinity of RFC for PCNA could be used by the cell cycle regulatory machinery to regulate DNA replication and other DNA metabolic processes.

## CONTROL OF PROCESSIVITY OF REPLICATIVE DNA POLYMERASES

The high processivity of the DNA polymerases that replicate the chromosome is achieved *via *their binding to sliding clamps. However, in recent years it has become clear that this interaction, rather than being static and permanent, could have a half-life substantially shorter than what could be expected from the measurements of processivity of the polymerases. In a series of elegant kinetic experiments using the phage T4 replication system as a model, it has been shown that each DNA polymerase molecule is replaced by another one at a rate of about every 10 seconds during chromosomal DNA replication (this would result in about 90 exchanges per replicated chromosome *in vivo*) [42]. The replacement of the DNA polymerases is efficient because the sliding clamp allows the simultaneous binding of at least two polymerases, which favors a quick engagement of the second on with the 3’-terminus of the DNA when the first one disengages from it. Thus, the term ‘dynamic processivity’ has been suggested to reflect the fact that the overall processivity of a reaction may depend on the enzymatic actions of a number of individual DNA polymerase molecules coordinated by the same sliding clamp [42, 43].

If an analogous mechanism of DNA polymerase exchange occurs in bacterial or eukaryotic systems remains to be demonstrated, but a related switch has been observed in the thermophilic euryarchaean *Pyrococcus abissy*. During chromosomal replication in this organism DNA polymerases belonging to different families take up successively the PCNA sliding clamp after it has been loaded on DNA by the clamp loader [44]. Pol D binds to PCNA left by the clamp loader (RCF) and extends the RNA primer. Then, Pol D is replaced by the replicative polymerase Pol B which contains a strong PCNA binding site. The switch is required because Pol B would not be able to extend from a RNA primer efficiently [44].

How do processive replicative DNA polymerases release from the sliding clamp? In *E. coli*, a mechanism for quick disengagement of DNA polymerase III (Pol III) from the clamp has been proposed when the lagging-strand DNA polymerase recycles to a new primer during Okazaki fragment synthesis, a switch that occurs every 2-3 seconds during chromosomal synthesis. How does the polymerase lose its tight grip on its β-clamp at the end of each Okazaki fragment? The alpha catalytic subunit of Pol III can make at least two contacts with the β-clamp, one internal (920-QADMF-924) and another one at the extreme C-terminus of the protein (1154-QVELEF-1159) [14, 15, 45]. As discussed in the previous section, both sites have been proposed to be important for processivity of the polymerase, although biochemical and crystallographic data suggest that the internal one could be better positioned for interaction with the clamp during elongation [45, 46]. The C-terminal site of alpha also binds tightly to another subunit of the replisome, the τ subunit of the gamma complex [47, 48], which regulates its processivity [49, 50]. The C-terminal domain of the τ subunit binds to single-stranded and double-stranded DNA [49]. It has been suggested that the τ subunit could compete with the β-clamp for binding to the C-terminal binding site on α. Indeed, in the presence of a primed template the affinity of τ for the C-terminus of α is low, but increases dramatically (>20-fold) in its absence or in the presence of double-stranded DNA [50]. Therefore during processive elongation the α subunit could be engaged with the β-clamp *via *C-terminal residues, which are then sequestered by τ when the complex reaches the double-stranded DNA of the downstream Okazaki fragment and τ-C ‘senses’ the absence of ssDNA. This DNA-sensing effect can be observed with a minimal system of just τ-C and a synthetic peptide containing the C-terminal 20 amino acids of the α subunit, indicating that these are the minimal players in a ‘processivity switch’ underlying the dissociation of α from the sliding clamp [50]. Recent crystallographic data obtained by the Steitz laboratory, however, have challenged this view and suggest that it is the C-terminal domain of the α subunit itself, which contains an OB fold which selectively only binds to ssDNA [46], could sense the nature of the DNA substrate and, *via *the τ subunit a conformational change on α, induce the processivity switch [46]. Although speculative given the absence of structural data for the τ subunit (or the highly flexible C-terminal region of the α-subunit of Pol III), these two scenarios hint at possible mechanisms by which a processivity switch could be established. Given the complex and dynamic nature of the interaction of Pol III with the sliding clamp, however, much remains to be discovered about the interactions of these proteins.

The eukaryotic replication fork has not been explored in sufficient detail to analyze the binding and release of DNA polymerases from the sliding clamp PCNA. A recent study showed that perhaps an intrinsic conformational change on DNA polymerase δ allows it to sense the double stranded DNA and release itself from the DNA to jump to the next primed template during Okazaki fragment synthesis [51]. This ‘collision release’ model, however, was tested on a highly simplified system and critical effects of other subunits of the replisome on the processivity of the polymerase cannot be ruled out.

It should be noted that, in addition to the moving platform that the sliding clamps provide, additional scaffolds are provided in the replisome in which to which DNA polymerases attach themselves. In *E. coli* a second scaffold is provided by the provided by the γ/τ-complex which coordinates the actions of the leading and lagging DNA polymerases [52]. On a different system, phage T7 DNA polymerase uses the factor thiorredoxin instead than a sliding clamp to substantially increase its processivity but in addition it binds to a DNA helicase in order to remain associated to the replication fork [53-55]. In this way several T7 DNA polymerases can switch at the fork by simply alternation of those already bound to the DNA helicase [54]. It is remarkable how nature has found analogous solutions to the problem of fast recruitment of polymerases to the active site by binding to multi-attachment site complexes within the replisome, be them in the form of sliding clamps, the γ/τ-complex (clamp loader) or a DNA helicase.

## TRANSLESION SYNTHESIS AND THE EXCHANGE OF DNA POLYMERASES

The critical role of sliding clamps in replication and repair has been highlighted in recent years with a large number of studies centered on the switch that occurs when a highly processive replicative DNA polymerase encounters damaged DNA that blocks its progression (reviewed in [56]). Nature’s solution to this problem is to allow the transient action of a specialized polymerase that can overcome the damage (translesion synthesis, or TLS). These polymerases have less stringent active sites that allow them to accommodate various types of bulky lesions and therefore are often much less accurate (low-fidelity) than replicative DNA polymerases. TLS polymerases display common features and structural elements, and they all belong to the Y-family of polymerases. The mechanism by which TLS polymerases take over extension of a primer 3’-end from a replicative DNA polymerase is based on their relative affinity for the sliding clamp.

In *E. coli* the Y-family polymerases Pol IV and Pol V act when Pol III stalls at a replication fork. Pol IV binds to the β-clamp at the same position on the clamp as Pol III [15, 57] but since the β-clamp can accommodate two ligands at the same time, both polymerases can simultaneously bind the same sliding clamp [58]. The mechanistic basis of this reaction was evidenced by the determination of the structure of a domain of Pol IV bound to the β-clamp [22]. The complex showed that Pol IV could interact with the sliding clamp *via *a highly flexible hinge that allows the protein to bind to the clamp in two distinct conformations: one extended in which Pol IV does not interact with DNA and would not clash sterically with Pol III bound to the same clamp, and one in which Pol IV would bend over DNA, take over the 3’-end from Pol III to extend the damaged template. Once the damaged DNA block is overcome, Pol III takes over again the 3’-end to resume high-fidelity, processive synthesis [58]. This model has been further supported by recent crystallographic work of the full-size *Sulfolobus solfataricus* Pol IV bound to its cognate clamp [23]. The detailed biochemical requirements also have been extensively studied in the case of *E. coli* Pol V (UmuCD), and reconstituted *in vitro* [59, 60]. Pol V also binds to the β-clamp in its hydrophobic pocket [15, 61] but there is no structural additional data about this interaction.

**Fig. (2):**
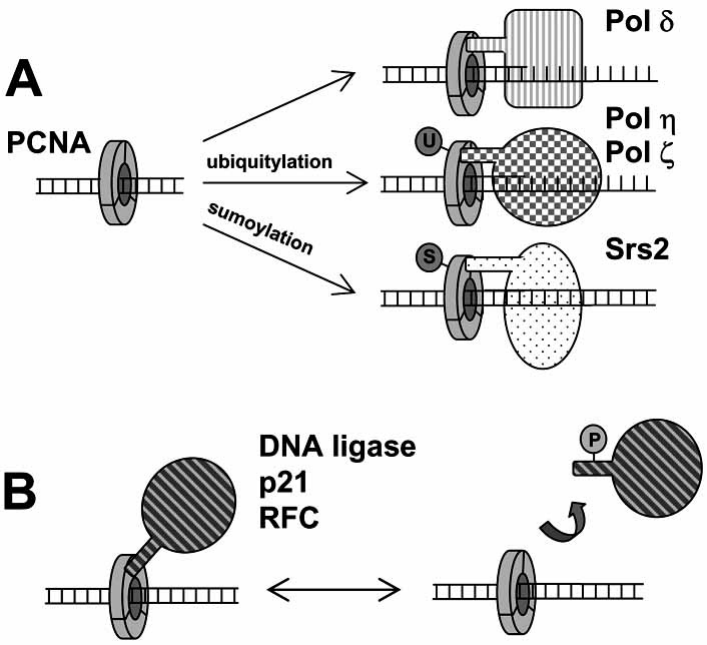
Two modes by which the interactions between eukaryotic sliding clamp PCNA and its partner protein ligands can be regulated. **A.** Ubiquitylation (U) or sumoylation (S) of PCNA leads to changes of affinity for DNA polymerases and, in the case of sumoylation, the recruitment of helicase Srs2. **B.** Phosphorylation of the ligand leads to a reduced affinity for PCNA (see Text). Phosphorylation often occurs in regions close to the binding site to PCNA.

In eukaryotes a higher level of regulation of binding to sliding clamps is based in the transient covalent modification of PCNA by sumoylation [62-64] or ubiquitination (Fig. **2A**) [62]. Posttranslational modifications have been observed in up to three residues on the surface of PCNA. In yeast PCNA residues K164 and K127 can be sumoylated [62], leading to recruitment of the helicase Srs2 inhibition of recombination ahead of the fork [65]. Ubiquitination of K164 of yeast PCNA by Rad6/Rad18 in response to DNA damaging agents leads to a reduction of affinity for Pol δ or Pol ε and probably of other proteins that bind to PCNA *via *the canonic motif, like the cohesion factor Eco1 or RFC1 [66]. On the other hand, ubiquitination leads to the recruitment of Pol η and Pol ζ [63] and Rev1 [67]. Ubiquitin needs to be removed from PCNA before Pol δ can resume processive DNA synthesis, a function performed by USP1 [35]. Finally, PCNA can be polyubiquitinated at K164 by Ubc13/Mms2/Rad5, but the function of this modification is unknown.

## PROCESSING OF OKAZAKI FRAGMENTS

The processing of the RNA/DNA hybrids generated in lagging strand DNA replication, also known as Okazaki fragment maturation, requires the concerted action of one or more nucleases, a DNA polymerase and a DNA ligase [24, 68, 69]. In *E. coli* this process is achieved by DNA polymerase I, which contains a 5’-3’ exonuclease activity within the molecule that digests the RNA primer, and by DNA ligase. Both of these enzymes bind to the β-clamp [70, 71] but it is unknown if their actions are coordinated by it. On the other hand, in the archaeon *Sulfolobus solfataricus *the DNA ligase, the nuclease Fen1 and a DNA polymerase can bind with distinct affinity to each subunit of the heterotrimeric PCNA present in this organism, showing that simultaneous binding to the sliding clamp could be possible and suggesting tight coordination of these enzymes *via *PCNA [6]. In eukaryotes numerous recent studies have highlighted the role of PCNA in coordinating the activities of Pol δ and Fen1 during lagging strand DNA synthesis [24, 72, 73].

Crystallographic studies of Fen1 bound to PCNA found that the interaction between these proteins is highly flexible [24, 25]. Fen1 binds to PCNA *via *an extended C-terminal peptide which at its base contains a hinge region that can adopt various conformations. Fen1 opens the DNA helix, enforcing a kink that facilitates flap recognition [24]. Multiple conformations of Fen1 could allow other proteins to access the DNA while Fen1 is still bound to the clamp because Fen1 can swivel on the hinge region next to the PCNA binding interface [25]. The effectors of the Fen1 switch from inactive to active conformations are unclear, but a kinked DNA structure is a likely candidate [24, 74].

The interaction of DNA ligase with PCNA and with nicked templates has also been extensively investigated functionally and structurally [75]. DNA ligase is a modular enzyme that can adopt open and closed conformations around DNA, as observed for human Lig1 in complex with DNA [76]. These large conformational changes could coordinate the action of DNA ligase with other PCNA-binding proteins [26], but it is unclear how binding to PCNA stimulates the ligation reaction. It has been suggested that perhaps PCNA somehow rigidifies the enzyme or allows it to distort and kink DNA [76], as observed for Fen1 [24]. In the closed conformation DNA ligase forms with PCNA two stacked rings with extensive contacts, and this perhaps would preclude other proteins from binding to the clamp. Perhaps this is related to the fact that ligation of DNA is typically the last step in Okazaki fragment processing or DNA repair reactions, and no subsequent binding to PCNA by other factors is required [26, 75, 77].

It is unclear how PCNA coordinates the handoff of the enzymes implicated in Okazaki fragment processing. DNA structure could dictate the various conformational switches that must take place for the three enzymes to perform their functions, but the mechanistic details are unknown. Given the extensive contacts made by these enzymes with the sliding clamp [24, 26, 27], it is interesting to consider the question of how the different ligands sever their binding surfaces to the clamp. As in the case of translesion synthesis, protein modification seems to be the answer. Fen1 is phosphorylated by PCNA-bound Cdk1-Cyclin A in late S-phase, leading to its release from PCNA [78]. Likewise, human DNA ligase phosphorylation in multiple sites is cell-cycle dependent and results in its release from replication sites, but the effects of this modification on PCNA affinity are unclear [79-81]. Interestingly, an interaction between human DNA ligase I and the clamp loader RFC has been reported which inhibits ligation, but this effect is reduced by binding of DNA ligase to PCNA [82]. Analysis of the binding surface of yeast PCNA to yeast Ligase I (Cdc9) showed that RFC could compete for the same binding surfaces [27], suggesting a complex interplay between these proteins. One possible explanation for the interaction between DNA ligase I and RFC is that RFC could be required to unload PCNA once DNA has been sealed. Since DNA ligase occludes the face of PCNA that RFC binds, an initial contact between RFC and DNA ligase would become necessary [82].

## MODULATION OF DNA REPAIR REACTIONS: MISMATCH REPAIR

The critical involvement of sliding clamps on DNA repair pathways has been well documented, where they act to orchestrate the enzymatic activities required for DNA damage recognition and processing. The mismatch repair pathway corrects errors introduced by the replication machinery and it has received intense scrutiny in the last decade [83]. The sensors of DNA mismatches, the MutS in *E. coli* or the MSH proteins of eukaryotes, can interact with the β-clamp or PCNA, respectively [70, 84]. In the case of MutS two interaction sites have been proposed: a weak one at the N-terminal, mismatch-binding, region of the protein, and one strong at the C-terminal region of the protein [14, 85, 86]. Recent studies of the *Bacillus subtilis* MutS-β-clamp interaction show that that β-clamp promotes the stabilization of MutS at mismatches *via *the C-terminal binding site, suggesting that mismatch recognition and β-clamp binding are tightly coupled to target MutS to active replication sites [86]. On the other hand, the binding site of eukaryotic MSH3 or MSH6 to PCNA is located at the extreme N-terminus of these proteins [84, 87-89]. It has been shown that PCNA could stimulate the preferential binding of MSH2-MSH6 to DNA containing mismatches [87], but recent structural data of the PCNA-binding domain, which shows this domain as highly flexible, seems to argue against this idea [88-90].

*E. coli*’s MutL and its eukaryotic homolog MLH1 also interact with the β-clamp and PCNA, respectively [91, 92]. MutL is a multifaceted protein which acts as a ‘matchmaker’ among the mismatch repair factors and that, as revealed by crystallography, can undergo major conformational changes [93, 94]. The interaction between MutL and the β-clamp seems to require binding of MutL to single-stranded DNA, a natural ligand of MutL [85, 94]. In addition, the interactions of MutL to the β-clamp and MLH1 to PCNA have been tentatively mapped using peptides to an internal loop (Loop 2) that undergoes a conformational change during the ATPase cycle of this protein [85, 94]. This loop is exposed to binding to the sliding clamp in the nucleotide-free MutL, but buried inside the N-terminal domain of MutL when the protein is bound to ATP, suggesting a possible on-off switch for the interaction [94]. The function of these contacts is unknown, but they point towards a highly dynamic interaction in which protein affinity is ultimately modulated by DNA structure and ATP-induced conformational changes. On the other hand, a recent study has shown that yeast PCNA is essential in orienting the endonuclease activity present in MLH1-PMS1 complexes to discontinuous strands of DNA [95]. While the role of sliding clamps in mismatch repair is undisputed, there is still no clear view as to their function within this pathway. The suggestion has been made that perhaps the mismatch repair enzymes operate in very close proximity or even within the replisome itself, and that their main anchor is the sliding clamp.

## FUTURE PROSPECTS

From the point of view of organic chemistry synthesis, the replication of a chromosome is an extraordinarily complex event. Human cells replicate their entire genome (3x10^9^ base pairs) in just a few hours and with an error rate of 10^-9^ per base pair. Hundreds of millions of years of evolution have perfected the biosynthetic machinery by adding, from bacteria to the vertebrates, successive and ever more sophisticated layers of complexity. Ultimately, however, orchestration of the myriad biochemical events that take place during DNA synthesis relies on sequential and finely-tuned protein-protein and protein-DNA interactions of specialized protein modules that perform particular functions in an orderly, thermodynamically favorable, manner. How is it ensured that only the required enzymes coordinate their functions and activities at the replication fork at a given time and place? Part of the answer is given by the observed ability of different enzymes to organize themselves on proteins that accept multiple ligands and act as scaffolds for sequential enzymatic reactions. Within the replisome, at least three such scaffolds have been suggested in the literature: sliding clamps, the sliding clamp loaders [43], and the DNA helicase [54]. Of these, the sliding clamps show the highest versatility and universality. An understanding of what governs attachment or detachment from the sliding clamps is therefore a critical aspect of the study of the multi-enzymatic reactions that drive DNA replication and repair. As reviewed here, cells employ a variety of means to modulate affinity for clamps, but in most systems studied to date the detailed mechanism remains to be analyzed (Table **1**). In prokaryotic systems a combination of conformational changes in the enzyme, ultimately brought about by specific DNA structures and coupled to ATPase activity, seems to be a widespread mechanism of controlling binding to sliding clamps. On the other hand, posttranslational modification of the interacting surfaces is the typical way by which eukaryotic systems modulate access of the various ligands to the PCNA ring. It even has been suggested that these modifications in PCNA could serve as a way to ‘reset’ the clamp and prevent it from binding any factor [66]. A field that requires more investigation relates to how are clamps removed from DNA, as we don’t clearly know what factor or factors are responsible for this process. The clamp loader (γ-complex or RFC) could be responsible or, as it has been proposed, one of the newly identified variants of RFC [1].

The diversity of proteins that interact with the β-clamp and PCNA makes sliding clamps one of the most trafficked elements in the cell protein network. Future work is likely to focus on the solving of additional crystal structures of complexes of sliding clamps and their partner proteins, perhaps in combination with relevant DNA structures. The analysis of the architecture of the ternary complexes of sliding clamps bound to active enzymes engaged with their natural substrates should be the goal because it is probably different in the absence of DNA substrate. Detailed analysis of affinities in the case of competition between clamps and other factors for binding to common sites on partner proteins will likely add to our understanding of how sliding clamps are regulated.

## Figures and Tables

**Table 1:** Documented Mechanisms that Regulate Binding and Affinity to Sliding Clamps

**Direct displacement by peptides or other proteins**	
- Replication arrest in *Staphylococcus aureus* by phage-derived peptides - τ processivity switch in *Escherichia coli*	[96] [45, 46, 49, 50]
**Conformational changes on the sliding clamp binding protein**	
- γ-complex (clamp loader ATPase cycle and DNA binding) - MutL (modulated by ATP and ssDNA binding)	[28, 29] [85]
**Phosphorylation of protein**	
- p21 (inhibition of binding to PCNA) - DNA ligase (effects unknown) - Fen1 by PCNA-bound Cdk1-ciclin A (inhibition of binding to PCNA) - RFC1 by cell-cycle kinases (inhibition of binding to PCNA)	[20] [79, 80] [78] [40, 41]
**Ubiquitination of protein**	
- Cdt1 (leads to proteolysis and prevention of re-replication)	[97]
**Sumoylation of PCNA**	
- on K164 or K127 (recruitment of the helicase Srs2 and inhibition of recombination ahead of the fork)	[62, 65, 66]
**Ubiquitination of PCNA**	
- on K164 by RAD6 pathway (trans-lesion synthesis by DNA polymerases Pol η and Pol ζ)	[62, 63]
**Polyubiquitination of PCNA**	
- on K164 by Ubc13/Mms2/Rad5 (trans-lesion synthesis regulation)	[62]
**Phosphorylation of PCNA **	
- Tyr211 by EGFR (stability on chromatin)	[98]
